# Drug Use as Boundary Play: A Qualitative Exploration of Gay Circuit Parties

**DOI:** 10.3109/10826084.2011.572329

**Published:** 2011-06-21

**Authors:** Patrick O'Byrne, Dave Holmes

**Affiliations:** School of Nursing, Faculty of Health Sciences, University of Ottawa, Ottawa, Ontario, Canada

## Abstract

Research findings have revealed that gay circuit parties may be locations that are disproportionately responsible for the increasing rates of many STIs/HIV among gay/bisexual men. Theories have been put forth that this may be the case because circuit parties are locales of prevalent drug use and unsafe sex. To explore the relationship between these two phenomena, in-depth qualitative interviews were undertaken with 17 men who (1) have sex with other men, (2) attended gay circuit parties in Montréal, Canada, in 2007. These revealed that drugs (including alcohol) were used intentionally to engage in unsafe sex, and then to justify this behavior after the fact. This process we called boundary play.

## Résumé

**La consommation délibérée de drogues comme prélude à des pratiques sexuelles à risque : une exploration qualitative des *gay circuit parties*:**

Les résultats de recherches actuels soutiennent que les « gays circuit parties » sont des environnements propices à la transmission des ITS et du VIH chez les hommes gays/bisexuels. Certains chercheurs ajoutent que la prise de drogues et les pratiques sexuelles à risque sont des comportements courants dans ces environnements. Afin d'explorer les relations entre ces deux comportements, 17 entrevues qualitatives ont été conduites auprès de 17 hommes, ayant des relations sexuelles avec d'autres hommes, et ayant participé en 2007 à des « gay circuit parties » à Montréal (Canada). Nos résultats montrent que les drogues ont été utilisées dans le but précis d'avoir des relations sexuelles à risque. Ce résultat de recherche est en contradiction avec le discours scientifique actuel qui sou-tient que la prise de drogues peut mener accidentellement à des pratiques sexuelles à risque.

## Resumen

**El uso deliberado de las drogas como un preludio a las conductas sexuales de riesgo: una exploración cualitativa de los “gay circuit parties”:**

Los resultados de las investigaciones actuales sostienen que los “gay circuit parties” son entornos propicios para la transmisión de las ITS y del VIH entre hombres homosexuales/bisexuales. Algunos investigadores han sug-erido que el consumo de drogas y conductas sexuales de riesgo son comunes en estos ambientes. Para explorar la relatión entre estos dos comportamientos, 17 entrevistas cualitativas se llevaron a cabo con 17 hombres que tienen relaciones sexuales con hombres, y han participado en 2007 a “gay circuit parties” en Montreal (Canadá). Nue-stros resultados muestran que las drogas eran utilizadas para el propósito específico de tener relaciones sexuales sin protectión. Este resultado de la búsqueda está en contradictión con el discurso científico actual que sostiene que el consumo de drogas puede accidentalmente llevar a prácticas sexuales de riesgo.

## INTRODUCTION

As a starting point, because both gay circuit paries (GCPs) and raves manifest some similarities, the latter type of party can be used to situate the former type of party. For example, they both typically take place in large venues and have repetitive and loud, “drum 'n bass” music played at a fast tempo in conjunction with intricate and elaborate light and laser shows. In addition, at both GCPs and raves, huge crowds dance, and often consume drugs—a fact that situates them as assemblies of nonmainstream participants and practices. Please note, in this context, alcohol was described, and thus coded, as a drug.

Beyond these similarities, however, raves and GCPs differ quite significantly: unlike raves, GCPs were created and denned in relation to the celebration of/by a particular sexual orientation. This means that while raves and GCPs are both parties that have been created by nonmainstream populations and both involve music, dancing, and drug use, the two nonmainstream populations are markedly dissimilar. GCPs were designed by and for a subset of men who have sex with other men (MSM). Raves, in contrast, seem to attract a more undifferentiated, nonmainstream group and are primarily attended by younger, often heterosexual, youth; GCPs are most often attended by 20- to 40-year-old, self-identified, gay and bisexual men who are above average in education and socioeconomic status. In addition, drug use differs: ravers prefer ecstasy; GCP attendees use a wider variety of drugs, including ketamine, crystal meth, gamma-hydroxybutyric acid (GHB), amphetamines, ecstasy and alcohol. These differences thus raise questions about the validity of using data about raves to understand GCPs.

In fact, the small quantity of research that addresses GCPs has often identified the participants as a distinguishable subculture, which is different from other dance-drug-party cultures. This suggests that many of the descriptions about drug use that were derived from other populations may not be valid for explaining the drug-using practices of GCP participants. Compounding the severity of inappropriately conflating GCPs and raves is that research (Ghaziani & Cook, 2005; Kurtz, 2005) suggests that GCPs may be disproportionately responsible for the recently observed increases in HIV rates among MSM.

On the basis of the foregoing points, the purpose of this research was thus to revisit the notion of drug use within the contexts of GCPs to determine how these activities in this specific milieu could be understood from a non-postpositivistic, nonpsychoanalytic perspective. The goals of doing this were twofold. On the one hand, the intention was exploratory: to see what results two researchers who are guided by the theories of Deleuze (O'Byrne) and Foucault (Holmes) would produce about GCP-related drug/alcohol use. On the other hand, the aim was to use the information that arose from such a nonmainstream position to inform HIV prevention work. This latter goal arose because while the traditional explanations of these phenomena are empirically based, rigorously developed, and logical in nature, they do not seem to be yielding the information that is needed to develop successful HIV prevention initiatives. Indeed, HIV rates continue to rise among MSM, and some authors (Ghaziani & Cook, 2005; Kurtz, 2005) suggest that GCPs and their associated practices are some of the possible reasons for this situation. As such, it seemed logical to explore this topic from different perspectives to see what information would arise (or, more accurately, be produced), while simultaneously delving into the larger task of identifying how this information could be used to produce novel HIV prevention initiatives for this target population. Regrettably, this second goal is not addressed within the scope of this article.

Therefore, because of the lacunae in the knowledge about GCPs and their potential role in HIV transmission, we undertook a qualitative, exploratory study to understand drug use from the perspective of the GCP attendees by means of direct observation of two circuit parties, questionnaires, and in-depth, semi-structured interviews. In this article, however, only the data from the 17 interviews will be presented because such a narrow focus allows for more in-depth discussion of the precise insights that the research participants revealed about drug use within the context of GCPs. Following this, the data will be explained using the concept of boundary play.

## CONCEPTUAL FRAMEWORK: BOUNDARY PLAY

To ground our explanation of drug use at GCPs, we employed the concept of boundary play, which neither initially described in relation to drug use at GCPs, nor originally called boundary play. Moreover, this concept was adopted during data analysis, not beforehand. In other words, while we were aware of the concept edgework prior to data collection/analysis, it was not originally included in the theoretical framework, which guided this work. Instead, it was incorporated as data analysis ensued because this idea provided an excellent structure for explaining our results. However, this necessitated that the concept of *edgework* be described as *boundary play* because this new term better reflected the scope, nature, and details of the collected data.

Boundary play can best be understood as a process during which individuals navigate a variety of edges, or “play” with various boundaries including the limits between sanity and insanity, legality and illegality, safety and danger, chaos and order, and life and death. The boundary is the dividing line between two opposing states of existence, and playing with such boundaries is the act of approaching or treading on these lines. An essential component of such acts is that in flirting with danger, individuals must demonstrate the ability to safely navigate perilous edges, boundaries, or limits without falling off/over the edge. During boundary play, individuals must be ready and able to negotiate and navigate extreme situations rapidly despite the odds being against successfully doing so. They must be able to avert the extreme and often irreversible loss, damage, or destruction to which they have exposed themselves.

In short, individuals who engage in boundary play are driven to what some may call extremes. In saying this, however, the term extreme must be nuanced. It is not a point that is too far, but rather the maximum point, or apex. It is the furthest point that one can reach without being/becoming unable to return and while such practices may seem self-destructive, this is not the case. Boundary play does not occur as a manifestation of inherent desires for self-harm. Instead, the behavior is seemingly paradoxical and manifests the desire to remain safe within otherwise dangerous situations that one has intentionally entered. Thus, boundary play is not an act of recklessness, a method by which to commit suicide, or a sign of personal disregard. Ultimately, it is not a manifestation of underlying psychopathology. Instead, it is the expression of pure desire.

## METHODOLOGICAL CONSIDERATIONS

### Design

This project was undertaken as a qualitative-based, exploratory research into sexuality and drug use and gathered information about history, culture, gender, etc. As part of this, attention was paid to the environment, social interactions, and the culmination of all the physical and nonphysical connections that produced the overall ambience and experience of the GCP. Data collection occurred through direct observation, autoadministered surveys, and formal interviews. As noted earlier, only the interview data results will be discussed in this article.

### Participant Recruitment

Recruitment for this study was not restricted to the target GCPs; it also occurred via posters (with a phone number and an e-mail address printed on them) in bathhouses, gay bars, clubs, and gyms, and sexual health clinics in three of Canada's biggest cities, which host the largest GCPs in Canada and have the largest urban Anglophone and Francophone populations. In addition, snowball sampling was used—interviewees were given the researcher's contact information, and asked to pass it on to other individuals whom they believed would be interested in participating. This recruitment method has been shown to be effective for *infiltrating* a group that engages in marginal practices (Platzer & James, 1997). The inclusion criteria were as follows: self-identifying gay or bisexual man who attends GCPs, has/does use(d) drugs at GCPs, and engages in sex with partners at or from GCPs. All potential participants were screened prior to meeting and only those who met all three criteria were formally interviewed.

### Data Collection: Formal Interviews

The principal data collection method was formal interviews, which occurred in the offices of the research team. During these interviews, participants first completed a self-administered questionnaire in which they reported their sociodemographics and sexual/drug use behavior. This information was obtained to gather a rich description of the interview sample. Following this, the participants took part in a taped, in-depth, semi-structured, open-ended interview, which lasted approximately 1 hour. For this process, a feminist approach to interviewing was used, with its corresponding engagement in emotional issues and development of trust. This personalization of the interview process helped redefine the interview context and equalize the power differential between the interviewer and the interviewee, thus reducing the power disparity and the unidirectional structure present in the standard interview (Fontana & Frey, 2003). This also allowed the interview to wander into areas that the interviewees chose, and it continued until the interview material no longer provided new information.

### Data Analysis: Epistemological and Step-by-Step Considerations

The analytic methods that were employed as part of this study could most readily be called a schema, or latent content, thematic analysis. This means that the analysis of the interview data occurred with the goal of unearthing the underlying *latent* content of the participants' interview data. However, this should not be understood in a psychoanalytic sense. Instead, as the underlying theoretical orientation of the authors who undertook this study is ultimately Deleuzian (O'Byrne) and Foucauldian (Holmes), latent analysis is not aimed at understanding the inner workings of the human mind, but rather is focused on identifying the structures of desire and power, which permeate both the undertaking and the description of a given event/phenomenon. Furthermore, such theoretical orientation also sheds light on the epistemological perspective within which this project took place—a poststructuralist orientation in which reality is the outcome of various competing discourses, interpretations, and interactions. An awareness of such information is imperative as one shifts through the data analysis and discussion, which follows shortly because it situates the constructivist method of data interpretation that occurred.

Such a theoretical orientation also explains another idea that underpins this research: the notion that researchers cannot remove, eliminate, or even bracket their personal assumptions, beliefs, or values (what some might call, their “biases”). As such, while it is stated earlier that the conceptual framework of *boundary play* was selected during the data analysis, this should not be understood to mean that this conceptual understanding *emerged* from the data. Indeed, to use such language is to deny the active process that occurred as part of this analysis: the interpretation and scrutiny that ensued according to the researchers' underlying life histories. The after-the-fact selection of boundary play thus occurred because both authors came to the conclusion that it encapsulated the points that the research participants raised. Nevertheless, owing to slight inconsistencies (as are noted earlier in the section describing the conceptual framework), this concept was changed slightly from edgework to boundary play.

*Steps of Data Analysis.* The specific methods by which this thematic analysis occurred were as follows. First, the authors familiarized themselves with the data through multiple readings of the interview transcripts, and then by engaging in numerous discussions with one another about the meaning and significance of the data. This involved repeated reviews of the interview data material to reflect and validate our initial interpretations. Second, and not necessarily a distinct phase from the first step, the authors began to generate a list of initial codes. Because this thematic analysis was latent content, or schema-based, it involved the identification of metaphors within the text. Codes were written directly into the margins of the printed transcripts, and were also compiled in an excel spreadsheet. At this point, the codes were not ranked, sorted, or filtered. Instead, they were simply listed along the vertical axis of the spreadsheet. Along the horizontal axis, each interview participant was listed, and a corresponding mark was made to indicate that this participant had mentioned the content of this code. As part of this mark, information was included to the line numbers of the transcript to: (1) verify the empirical data from which these codes arose at a later point and (2) to ensure a strong audit trail. As this second step began to finish, the third phase started: the aggregation of these codes into themes. This was the step wherein similar codes were identified and placed together under a unifying heading. Thereafter, the fourth step started, which involved reviewing the themes by returning to the actual coded interview material to ensure that the codes did in fact belong together. Here, we also sought to ensure that the themes were both internally homogenous (i.e., the codes that had been combined within each theme were sufficiently coherent) and externally heterogeneous (i.e., themes were identifiably distinct from one another). As the final phase, the themes were defined and named. This involved clearly and concisely articulating the content of each theme, both as an identifiable topic and in relation to the other themes. This was the process of producing each theme's narrative and the overall story of the themes as a whole. At this point, the written text that arose from this process was transformed into formalized manuscripts.

### Drugs and Its Users: Understanding GCP-Related Drug Use

In total, 17 interviews were carried out. Each interview participant also completed a self-administered questionnaire regarding their age, ethnicity, socioeconomic status, and sexual and drug-using practices. These data are presented in the following sections.

### Sample Description

In order to provide a detailed overview of the sample, a series of descriptors have been presented in Table 1. To summarize, the average reported age of the participants was 36.3 ± 9.4 years; English was reported as the first language for 76.5% of the sample; 88.2% reported that their sexual preference was exclusively for men; 76.5% of the sample had at least college education; 88.2% were Caucasian, and 70.6% of the sample reported a gross annual income of at least $30,000 Canadian Dollars (CAD)—note that for the year 2006 (when data collection occurred), the Canadian low-income cutoff for the cities where data collection occurred was $21,202 (Canadian Council on Social Development, 2006).

**Table 1:** Socio-economic data

	Mean Median	Frequency	Percent	Cumulative percent
Age	36.3 ± 9.4 yrs.			
	36.5			
Language				
English		13	76.5	76.5
French		4	23.5	100
Sexual preference				
Men		15	88.2	88.2
Men & women		2	11.8	100
Education level				
No diploma		1	5.9	5.9
High school		3	17.6	23.5
College		4	23.5	47.1
Bachelors		5	29.4	76.5
Masters		2	11.8	88.2
Doctorate		2	11.8	100
Ethnicity				
Caucasian		15	88.2	88.2
African Canadian		1	5.9	94.1
Asian Canadian		1	5.9	100
Income (CAD)				
<$10,000		1	5.9	5.9
$10,000–$30,000		4	23.5	29.4
$30,000–$50,000		5	29.4	58.8
$50,000–$70,000		2	11.8	70.6
$70,000–$90,000		1	5.9	76.5
>$90,000		3	17.6	94.1
Missing		1	5.9	100

To add further details about this group, Table 2 reports the number of sexual partners the participants reported and HIV testing history and status. The mean number of sexual partners was 21.4 ±48.1, with a median of 7. This variability occurred due to the values ranging from 1 to 200, with 41.3% reporting fewer than 4.5 partners in the preceding 6 months. Considering past HIV testing history, all individuals had undergone a test, with only one individual not reporting the result of this test and one individual reporting that they had previously tested HIV positive. For sexually transmitted infection (STI) testing, 47.1% reported a previous STI, of which gonorrhea was the most common (*n* = 4).

**Table 2:** Sexual and STI/HIV testing practices

	Mean Median Min/Max	Frequency	Percent	Cumulative percent
Number of sex partners (in last six months)	21.4 ± 48.1			
	7			
	1–200			
Number of sex partners				
0.5–4.5		7	41.3	41.3
0.5–4.5		2	11.7	53
0.5–4.5		3	17.7	70.7
0.5–4.5		2	11.7	82.4
0.5–4.5		1	5.9	88.3
0.5–4.5		2	11.7	100
Previous HIV test				
Yes - status not given		1	5.9	5.9
Yes - status positive		1	5.9	11.8
Yes - status negative		15	88.2	100
No		0	0	100
Previously had an STI				
Yes		8	47.1	47.1
No		9	52.9	100
If previously had an STI, which one				
No response		2	25	25
Gonorrhea		4	50	75
HPV		1	12.5	87.5
Crabs		1	12.5	100

### Interview Data

Two main themes emerged from the interview data: (1) the participants used drugs to explore life to its limits and (2) the participants justified these explorations as the result of drug use when the outcome was deemed undesirable. Thus, the research participants noted that drugs both permitted them to explore and to excuse their explorations *ex post facto.*

*Theme One: Purposive Drug Use to Explore Life to Its Limits.* The research participants in this study readily identified their desires to explore. However, this did not mean overcoming boundaries. On the contrary, it involved an open-ended and experimental process without specific goals. The participants highlighted that such exploration occurred through, and as a result of, drug use, partying, and sexual conduct. The following participants illustrate this:

I guess in my twenties I was exploring, being young, and youthful, and partying, and sex, and all that. (Ott-7)

I tend to explore. I need to know. I mean, people say, “oh have you tried this?” “No, but I'll try once,” and then I'll know if I like it or not. (Ott-4)

The above quotations illustrate that exploration is a primary activity of the research participants. They wish to experience/experiment with many new sensations.

However, this exploration is not without limits. Instead, it can be described as the process of standing as close to an edge as possible, but without falling off. It is a highly regulated and controlled activity that involves reaching the limits of self-control, without exceeding them. The following participant clearly articulates this point about how pushing limitations gives him “everything,” but only up to a certain level. At this undefined point, the following participant realizes that he has gone too far, and stops pushing. Indeed, he decides that he is “not playing anymore.” He states:

What I was saying is that you're pushing; you have everything when you're pushing too far, but there's time when I'll say I'm not playing anymore. (Ott-14)

In the foregoing quotation, Ott-14 exemplifies what many other participants described: a process of approaching personal limits in order to explore them, but without going too far. At the point where his exploration does go too far, he states that that is the “time when [he'll] say [he's] not playing anymore.” In this quotation, that which stops him from “playing” is not reported—perhaps (and as will be suggested in the theme two), this is because it is only *post hoc,* after the exploration, that is, that he can explain what caused him to stop “playing.” Nevertheless, further investigation with Ott-14 confirms that his goal is to “push [him] self to the limit,” but not do destroy himself in the process. “Exhaustion is the goal,” not death, destruction, or devastation. The bounded nature of this “push[ing],” which we call “exploration” here, is of particular interest because it demonstrates that the participants (as exemplified by the text of Ott-14) did not intentionally desire their own demise. Indeed, they did not seem to be intentionally attempting to inflict harm upon themselves. Notice how Ott-14 explains this process:

I'd push myself to the limit of exhaustion.

But not destruction?

No. For example, three months ago, I was ready to die and I decided let's go in detox and just give yourself another chance, but I was ready to commit suicide, so I had at least that survival that keeps me. (Ott-14)

In this second statement, Ott-14 provides an example of his “push[ing]” (i.e., exploration) that exceeded the “limit of exhaustion”—indeed that went to the point of suicidal ideation. However, at the point where he has gone too far, a withdrawal occurs (“detox” and “that survival [instinct] that keeps me [alive]” kicks in). Our interpretation of this process is that both Ott-14 and the other participants who described such a process withdraw when their explorations cause them to surpass a certain point (i.e., their limits). Again, we posit that this may be because the goal is to flirt with danger, not to be destroyed. That is, pushing beyond personal limits represented a “risk” that this participant did not want to take. Ott-14 further illustrates how he would push until he was about to “lose everything,” but then draw back:

To the limit of losing everything, yeah, I would do it completely. And that's what I'm thinking these days is the excess. Now I have to rehabilitate myself in finding pleasure without excess, but I still need my highs. (Ott-14)

As revealed in this quotation, Ott-14's ability to experience pleasure seems to be inextricably linked with “excess” and pushing “to the limit of losing everything.” His desires, one could suggest, involve a form of bounded exploration, an investigation into everything that can be experienced within a given set of limits. The caveat, however, is that this participant also desires to retain control of himself by placing parameters onto his exploration.

However, no other participants reported such severe outcomes (detoxification or suicidal ideation) related to their substance use. In contrast, most participants reported that both GCPs and substance use served as the means by which desired experiences of exploration could be realized. In all of this, the sequence remains unaltered: the participants' aimed not to lose control, but rather to maximize personal experiences by reaching this limit. In the following two quotations, Ott-8 further demonstrates this process. He states:

So when we're going out to a [GCP name], we have a good time, and the energy levels rise and we just become more motivated, more willing to consume, and consume, and push our limits. (Ott-8)

Yeah, but, you know, I'm STILL able, when I'm drunk, to recognize my limits—what I'd do and what I wouldn't do. I think that kind of says that I still DO have limits when I'm smashed. (Ott-8)

For Ott-8, GCPs are locations where experimentation of life is permitted, where he has “a good time,” where his “energy levels rise,” and where he “just become[s] more motivate, more willing to consume. Most importantly here, GCPs are places where this participant reports that he and his friends “push [to their] limits,” but do so while still being cognizant not to surpass these limits.

For some participants, sexual practices represent a limit that can be explored by lengthening the duration of contact, trying new experiences, modifying a particular practice, or increasing the number of partners. Prolonged sexual escapades, for example, become a challenge, in part, to see if they are physically possible and to push pleasure to its limits. How much pleasure is possible? For the following participant, the performance of oral sex for prolonged periods is one method by which he pushes himself to his limits and achieves pleasure:

I can go to extremes … I like to do things to extreme. So, for a lot of guys, sucking for hours non-stop is not always the most comfortable thing, but I like to please someone, so having someone pleased, and enjoying what you're doing and enjoying what you're doing, is a turn on. (Ott-1)

Here Ott-1 reports that how he likes to engage in extended oral sex that is “not always the most comfortable thing,” but which transforms through its excess into “a turn on.” This practice is an intentional exploration of limits through drug use that allows for sexual practices and new pleasures. The following participant reports that he as well enjoys engaging in sex while under the influence of drugs because it allows him to intentionally explore the limits of his body, to “experience everything that [one] can experience.” He states:

You would rather have sex with drugs?

Yeah I prefer it with. It changes my limitations. I find I can get more into it with drugs than without. I'm more susceptible to suggestion. I will try new things, but things that I would normally not try. I want to experience everything that you can experience. I think it's the whole purpose. Try everything once; if you don't like it, don't go back. (TO-1)

For TO-1, drug use becomes important as a means by which the physical limitations of his body (i.e., fatigue, etc.) can be surmounted. This is echoed by the following participant who reported that ecstasy (E) consumption is one method of partying for “four days”—an experience that the human body cannot endure unaided. He states:

After four days of partying most people, unless they were popping E or something crazy like that, their energy lowers, their mood and the atmosphere starts to die down. (TO-1)

In the same vein, another participant relates that he uses ecstasy to offset fatigue:

[Because of fatigue], usually at around four o'clock in the morning it will probably be ecstasy and usually when you come down from ecstasy you use marijuana so you don't come down harder. (Ott-11)

In the previous two quotations, the participants push their bodies to their limits. This demonstrates the deliberate use of drugs to achieve a specific form of exploration—not too much to overdose, but not too little so that they feel fatigued. In the second quotation, Ott-11 reports using additional substances to dynamically counteract the effects of previously consumed substances—the “use [of] marijuana so you don't come down harder.” In effect, these two participants illustrate that, overall, their limits, while pursued and played with, must not be exceeded. In fact, sexual and drug use practices are undertaken with extreme calculation. For example, the following participant remains aware of his limits to ensure that the withdrawal period does not last for days:

I know what my limits are. I know when enough's enough. I know what time to take them. I never take them past a certain time, not take too much. I'm never up for like days on end doing that sort of thing. I'm very wary of that. (Ott-8)

This participant consumes drugs, but not to a point that he feels is excessive. His description of exploring his limit indicates an absolute measure that should not be exceeded. As previously noted, the analysis of the interviews identified that each individual possessed different limits, but to each participant, personal limits were reported as objective (“THE limit”): “But know what you're doing and know your limits, or know THE limits” (Ott-4). This quote demonstrates that despite Ott-8 's substance-induced explorations, his goal is to remain cognizant of his limitations at all times. There is, thus, a point that the participants do not wish to pass, but at times, accidentally do. In these situations, drugs serve a secondary role: they justify transgression.

*Theme Two: Drug Use as Justification.* In addition to deliberately consuming drugs to experience the effects that these substances can produce, the participants identified that these substances can also serve as an excuse for some of the actions they undertook while intoxicated. This means that while the participants used drugs to overcome some of their limitations (subtheme one), they also used these substances to justify their transgressions if they did not appraise them favorably once they had again become sober. In the following quotation, Ott-10 illustrates this process when he indicates that alcohol affects his decision-making processes. He states:

Sometimes you just don't have the strongest hold on yourself.

What do you mean by “the strongest hold on yourself”?

You make decisions that you wouldn't normally make if you were not drunk.

So when you're drinking, you do things that you wouldn't when sober?

Yeah. (Ott-10)

Here, Ott-10 notes that substance-induced intoxication causes him to “make decisions” that he “wouldn't normally make.” In making such a claim, Ott-10 is attempting to relinquish, or at least diminish, personal responsibility for his own actions. In effect, his statement can be interpreted to mean that Ott-10's sober self is unaccountable for his drunk self based on the rationale that alcohol diminishes one's ability to make sound, logical decisions, and thus this substance is the cause of people's behavior.

Other participants, such as TO-1, however, refute Ott-10's claim by stating that “if you know your own limitations, then you can't turn around and blame the drug for it.” TO-1 continues: “Get stoned so that you can do it [i.e., pursue one's desires], but don't get so stoned that you lose control” (TO-1). In both these quotations, TO-1 maintains the idea that drugs can cause a loss of control, but this loss of control is an extreme state; it is not the usual outcome. Indeed, TO-1 argues, as did many other participants, that the usual outcome of drug consumption is to render one more likely to pursue otherwise inhibited or repressed desires. When Ott-10's claim that alcohol (as a drug) diminishes the hold he has on himself is interpreted through the lens of TO-1 's statement, we can begin to understand that recreational drug use serves an important role in distancing the user's intoxicated self from his sober self.

Further exploration of this topic/idea with other participants revealed that the *ex post facto* relationship between alcohol and drug use and behavior is one of justification. Ott-11 reported that he uses drugs to justify the actions he undertook while intoxicated when he feels that he needs to. An example of such a situation is when he *blames* sexual contact with an unattractive partner on drug-induced visual impairment. He states:

It's not usually what you did, it's usually who you did it with. That's what they're referring to. It's like, “oh, I can't believe I took that monster home.” That's probably what it is, their vision was really impaired. I think that's what they mean by it. My mother can tell me that she doesn't like getting fucked, and I call her a liar, because it's a human bodily function that we enjoy. So, for getting fucked, it's referring to whom you've been doing it with. (Ott-11)

According to Ott-11, a person's claim, including his own, that specific sexual contacts would not have occurred without intoxication is only required when that individual perceives his actions to be unacceptable (e.g., sexual contact with an unattractive partner). In such cases, drugs serve a dual purpose: (1) to permit individuals to push their limits and (2) to absorb the blame if the outcome of such behavior is deemed inappropriate. By extending Ott-11's claim to its logical limit, we can interpret the idea that if one woke up to find an attractive person in one's bed, one would be unlikely to *blame* the situation on drugs. From the perspective of Ott-11's statement, if one were to engage a partner who continued to appear attractive after the drug-related visual impairment had passed, one's intent to satisfy what Ott-11 calls “a human bodily function that we enjoy” might be more openly acknowledged as intentional and purposive. In all other cases, however, Ott-11 suggests that substance use will continue to be blamed.

Ott-11's insight also helps to explain the seeming inconsistencies that arose in other participants' interview data, such as when they adamantly reported that drugs did not produce changes in their behavior, but then described situations in which the exact opposite occurred and drugs were the identified cause of their behavior. Ott-11's insight describes these incompatible occurrences as being isolated situations in which drugs are used to disassociate from outcomes that do not correspond with the values of the sober self. This only happens when, after the fact, the drug-related outcomes are deemed suboptimal. As an example of this, review the following five quotations from Ott-7, during which he switches from stating that drugs do not change his behavior to suggesting that drugs, because they “took his personality into darkness,” are at least partly responsible for his acquisition of HIV. He states:

In my twenties I was exploring, being young, youthful, partying, having sex, and all that. I was totally connected with all the circuit boys. I was young, buffed, and having a great time. (Ott-7)

My practices never changed in any way. There were times when I was so high that there could have been a slip, but even then, I was really careful. (Ott-7)

Well, it [i.e., drugs] guarantees fun. You look forward to going to the party, and picking up whatever beforehand, and planning your whole night out. And if it escalated beyond that, so be it, too. (Ott-7)

The HIV definitely happened to me during a party. Hundred percent. If it wasn't the time that I'd mentioned, it could have been a slip somewhere else, but it pretty much happened then. (Ott-7)

It [i.e., drugs] took my personality into darkness, into doing riskier things. It would just push me to the dark side, push me to do things that I would never do in a million years. Yeah, it turned me into, like, this demon. (Ott-7)

Here, Ott-7 describes what seems to be an inconsistent story: one in which drugs both *do* and *do not* change his behavior. In the first three statements, Ott-7 describes how he planned his nightly drug use, which might have included undertakings that were not part of his original plan. In the fourth quotation, Ott-7 identifies the point at which he suspects he became HIV positive, and then proceeds to discuss how drugs, due to their having taken his “personality into darkness, into doing riskier things,” were the reason that he engaged in the sexual practices that ultimately caused him to become HIV positive. As discussed, Ott-11's statement helps clarify that Ott-7 is likely using drugs as justification for an unwanted outcome. This reconciles the differences between his initial statements that drugs did not affect his behavior and his later assertions that drugs had taken him to “the dark side.”

Another example of dissociating intoxicated-self behavior from sober-self behavior can be seen when the following participant at first relates that he is a specific *type* of person (i.e., he outlines his sober self), then how alcohol (as the drug he uses) changes his behavior (i.e., his drunk self), and lastly, that his actions are actually planned. He states:

What type of partying do you usually do?

I usually just go out to the bar with friends. I've never gone to a bathhouse. I don't do that kind of partying. I go to a bar with friends, have a couple of drinks, maybe three drinks, dance, and go home at the end of the night. (Ott-8)

My friends were telling me that we had tequila a couple of weeks ago and I was just absolutely crazy, on the speakers and everything. It's kind of funny now that you mention it. I see myself TOTALLY smashed, and doing these crazy things: getting all touchy-feely and gropey. I'm not usually like that. I'm kind of holding back, and shy, and quiet, and stuff. It just seems that another person comes out. (Ott-8)

I think it's easier on me, self-esteem-wise: Let's just not have a plan and let's just see what's going to happen. If something DOES happen, sure, it'll be cool. I think it'll be more of a let-down though if I establish a plan, go to the bar, and the plan fails. (Ott-8)

In the three foregoing quotations, Ott-8 describes an inconsistency that is similar to the one reported by Ott-7. Ott-8 describes himself as being a specific *type* of person, then blames his out-of-character actions on substance use, and finally admits that the behavior he previously blamed on alcohol is actually part of a plan that he does not acknowledge. In the third and final excerpts, Ott-8 enhances our understanding of what Ott-11 states is the use of intoxicants as justification *ex post facto.* It protects the sober-self's self-esteem. To acknowledge that one knowingly made decisions that can be evaluated as unwise or illogical is to put one's intelligence and rationality in question. However, to pretend that these actions are unintentional to the point that they are the result of external agents (i.e., drugs) protects the rational/logical/intelligent sober self from its seemingly irrational desires. Thereby, drugs serve as both personally and socially acceptable excuses for behavior.

## DISCUSSION

A summary of the foregoing interview data reveals that the interview participants reported that they deliberately consumed drugs (which in this context includes alcohol) to push themselves to their limits and to excuse themselves, after the fact, for having done so. In other words, an in-depth analysis of the data revealed that the research participants were intentionally and actively involved in the exploration of their personal limits and boundaries—both in relation to approaching and retreating from them. This sequence of approaching and receding from limits via drug use corresponds quite evidently to our description of boundary play: the process of exploring, experimenting, and approaching one's limits without exceeding personal boundaries. The goal of the participants was to move toward the event horizon of their identities, to navigate this dangerous border, and then to return to their routine lives and selves unscathed. They wanted to explore and experience life, but without irreversible damage to their everyday life/self.

The first step in this process was the use of drugs to diminish fatigue, negate pain, and override psychological inhibitions while attempting to avoid any form of irreparable or irreversible harm. In each case, the result of drug use was an uninhibited expression of desire, an indulgence in sought-after pleasures—that which we called an exploration of life. That is, through the consumption of these substances, the research participants were able to approach the extreme limits of their usual parameters of behavior and self. Stated differently, these men were able to engage temporarily in a process of pure *becoming* without being—an exercise of opening themselves up to endless possibilities of change and movement (Deleuze & Guattari, 1980, 1987).

Once such thresholds had been reached or explored, however, the participants reported that they sometimes blamed these substances (after the effects of the drugs had worn off) for any activities that they had engaged in during their boundary explorations. This occurred, primarily, when they later evaluated something that they had done during their period of intoxication as antithetical to their ideations of a sober self. Thus, if these men determined that they had pushed a boundary too far, they would explain this occurrence as a consequence of drug/alcohol ingestion rather than one of personal desires, or a lack of self-restraint. In explaining their actions and practices in such a way, the participants positioned themselves as victims of their own intoxicated selves; they became the innocent casualties of the substances they consumed. However, further exploration of this point indicated that the use of drugs served more precisely as an excuse for behavior that an individual does not wish to take ownership of.

This latter point is of central importance in our understanding of GCP-related drug use among the men involved in this study. In fact, this second function of substance use guided our understanding of this practice as a form of boundary play. This was because the *ex post facto* use of drugs as excuses illustrated that the participants in the study were not attempting to destroy previous conceptualizations of themselves; rather they were, for a designated period of time (i.e., the duration of the ingested substances), seeking to explore everything that could be part of their existence, from the unknown and unexplored to the known, but apprehensively desired. This signifies that the participants in this study did not have desires for self or social destruction; they simply wanted to explore their boundaries.

When the two functions of GCP-related substance use are considered simultaneously, drugs can be understood as transport mediums—agents which both permit and excuse an individual's unconventional behavior. From a boundary play perspective, drugs allow the individual to move to the edge of their boundaries and then permit them to return to the safe, central zone of their behavioral limits. These substances transport their users from nucleus to fringe and then back again—a process wherein individuals carry out their desires to the limit, and then argue that such actions are the result of intoxicants and not personal desires. As part of this process, the intoxicated self is thus separated from the sober self.

The significance of these findings is that they illustrate a previously undocumented explanation of GCP-related drug use. More specifically, these findings add to what previous authors have clearly identified as an almost incontestable link between drugs and unsafe practices. For example, (1) Mattison, Ross, Wolfson, Franklin, and HNRC Group (2001), who, based on their nonrandomly distributed 1,169 three-minute surveys at three GCPs across North America between 1998 and 1999, identified that most GCP attendees attend GCPs to fulfill their desires for “community, enjoyment, and celebration” (p. 125); (2) Ross, Mattison, and Franklin (2003), who used the same data set as Mattison and colleagues (2001); and (3) Colfax and colleagues (2001), who, in their telephone-based survey of 295 men in San Francisco, all reported that GCP-related drug use strongly correlated with unsafe sexual practices. Mansergh and colleagues (2001), who used the same data set as Colfax and colleagues, also found this relationship between drug use and unsafe behavior, and Lee and colleagues (2003), who also nonrandomly administered surveys to 173 men on-site at GCPs, illustrated this quite explicitly: “MDMA [ecstasy] use was also associated with significantly more receptive anal intercourse” (p. 47). This quotation summarizes the findings of the previously undertaken research about the strong relationship between drug use and unsafe behavior at GCPs. The present study found similar results.

In addition to this similarity, however, the results of our study and those of the previously undertaken quantitative studies involving GCPs differ quite substantially—particularly in relation to the proposed explanations for the relationship between drug use and unsafe practices. Considering Mattison and colleagues' (2001) work, this difference relates to the interpretation of why the consumption of multiple drugs correlates with unsafe sex. In contrast to the findings presented in this article, Mattison and colleagues (2001) proposed that drug interactions render users more disinhibited and amnesic and this causes them to engage *unknowingly* in unsafe sexual practices. Note as evidence, the following quotation from these authors: “It is probably reasonable conjecture that not only is it likely that users of multiple drugs are less likely to be able to predict or control drug interactions but also that as the number of drugs used simultaneously increases, disinhibition and amnesia may increase” (p. 125).

However, on the basis of the actual data that these researchers collected, the explanations of Mattison and colleagues (2001) should be approached with caution because, on the one hand, they failed to collect the necessary data on sexual intent to make such assertions, and on the other hand, when data that specifically explored the relationships between drugs and unsafe sex were collected, these findings were refuted. Therefore, we suggest that Mattison and colleagues' (2001) theory about multiple drug consumption and unsafe practices is based on personal assumptions that no individual would intentionally engage in unsafe practices. In doing so, these researchers maintain what Bataille (1962) calls an outsider perspective—one in which practices are interpreted exclusively in relation to the biases of the nonparticipant observer. This is particularly relevant when Mattison and colleagues arrived at the earlier assertion after having claimed that “it is interesting that the reasons for party attendance appeared to also predict unsafe sexual behaviour, with attending 'to have sex,' to be 'uninhibited and wild,' and 'to look and feel good' all predicting higher levels of unsafe behaviour” (p. 124). Here, the seeming conflict in Mattison's research findings may be explained using the idea put forth in this article about substance use and boundary play.

Indeed, when in-depth qualitative interviews are used to explain the relationship between GCP participants' drug use and unsafe practices, the results reveal that many of the outcomes related to drug use are not necessarily unintended. These substances are part of a deliberate act of using drugs to engage in boundary play. In their foregoing quotes, the participants of this study clearly indicated that drugs do not change their behavior. Rather, consequences such as HIV acquisition or sex with an unattractive partner are unintended by-products of boundary play for which substance use can easily take the blame. Such a distinction, while seemingly inconsequential, may be of the utmost importance for frontline HIV prevention workers who wish to design effective HIV/STI prevention initiatives for this group. (This point is discussed further in the section on limitations and research/practice implications.)

Unfortunately, similar theories were created to explain substance use and unsafe behavior when Colfax and colleagues (2001) extended their analysis beyond the data they had gathered as part of their study. In doing so, these authors interpreted their data (which revealed a strong correlation between drug use and unprotected anal sex with a partner of either sero-unknown or sero-discordant HIV status) to mean that “drug use is influencing participants' decisions to have unprotected sex” (p. 376). They then proposed that drug use induces such outcomes because it makes “participants unknowingly engage in riskier behaviour than they would without drugs” (p. 376). However, a detailed review of Colfax and colleagues (2001) article reveals that they did not collect any data on preexistent sexual intent. Therefore, while their claims are based on empirical research, it may be that these authors incorrectly applied mainstream ideas about drug use to frame their explanations. Unfortunately, this practice, while able to produce solutions that may be valid in some situations, does not necessarily advance current understandings about drug use and its relationship to unsafe behavior at GCPs.

Moreover, in an almost identical manner, Ross and colleagues (2003) applied explanations about drug use that were developed based on groups other than GCP participants. This resulted in a declaration that drug use induces cognitive escape. Although this interpretation seems to resemble the research findings presented in this article, further inspection reveals that this is not the case. On the basis of the findings of our research, drugs are not used to escape because “escaping” inherently requires an overcoming of constraining boundaries. Escape is an act in which one “break[s] free from confinement or control” (New Oxford American Dictionary). The participants in our study did not describe using drugs to escape any form of confinement or control. In fact, for these men, escaping one's boundaries meant failing in their attempts at boundary play. If they escaped, and thus moved beyond their personal boundaries, these men, first, invoked the excuse that drugs were the reason for adverse or unwanted outcomes, and then “quit playing” as one participant described it, despite the fact that all participants reported that both drugs and alcohol do not fundamentally change their behavior. This reveals that for the men in this study, escape is not a valid explanation for their behavior. They wanted to explore, not escape, and to move within their boundaries, not overcome them.

Furthermore, in the qualitative literature on GCPs, deficit-based explanations were most often invoked to explain the occurrence of drug/alcohol use and unsafe behavior. Lewis and Ross (1995), the first researchers to address GCPs, for example, indicated that GCP-related drug use in Sydney, Australia, was a means by which the 17 GCP attendees who were interviewed escaped from the daily hardships and rejections of that gay men experience within a predominately heterosexual society—a seemingly valid point. However, this raises the question: Are these two authors suggesting that GCPs and their associated drug use would become obsolete if gay men were socially accepted? Kurtz's study (2005) involving four focus groups of 3 to 4 men for a total of 15 men raises the same question. He found that his research participants also used drugs to overcome negative emotions resulting from the difficulties of their daily lives.

Next, Westhaver wrote two articles based on an ethnographic study of 35 GCPs across North America between 1998 and 2002. On the basis of this extensive data collection, Westhaver (2006) first argued that risky behavior at GCPs occurs as a result of individuals wanting to gain a sense of recognition that “is immanent in, but lacking from, current heteronormative social conditions” (p. 366). Westhaver (2006) then argued in a second article that GCPs should be understood as bodily, not cognitive, experiences of empowerment wherein men can express their homosexuality without reproach. Although this second article is different from the first one, it too was predicated on the belief that risky behavior was the manifestation of underlying deficits. This is somewhat paradoxical because Westhaver (2006) stated in his first article that he rejected the idea that risky practices can be understood as signs of “irrationality or moral depravity” (p. 347). Nevertheless, in this article, the author returned to a deficit-based explanation of risky behaviour—just one that did not label risky practices as either irrational or morally impure.

Although the results of these other research projects differ from the results presented here, this by no means serves to diminish the importance or validity of these previously undertaken studies. In contrast, these comparisons were made simply to highlight the differences that were found in this research and in others. As for explanations about why such differences may have occurred, one must remember that, first, a different theoretical orientation was used in this project. This different perspective, while being highly critical, was also both accepting of illicit drug use and sceptical of the notion that unsafe sex is a pathological activity. As a second point, some of the differences that arose could also be explained on the basis of the methodological approaches that were used. This study was an in-depth qualitative study, which employed thematic analysis (described earlier), rather than a quantitative study.

In addition (and this point applies to the qualitative research as well), differences might have arisen because of the stated study goals. In this study, the explicit purpose was to expound as much detail as possible about the relationship between drug use and unsafe behavior—a goal that did not seem to exist in most of the other research projects. Thus, in contrast to the quantitative studies, which aimed to identify if a relationship existed between these two items, this study produced understandings about the relationship between drug use and unsafe behavior based on semidirected participant input, rather than from the imported use of findings from other, non-GCP-based qualitative studies about substance use and unsafe behavior. This, by no means, diminishes the importance of this previous research, as this previously undertaken quantitative research both justified and identified the need to undertake this project.

## LIMITATIONS AND HIV PREVENTION/RESEARCH IMPLICATIONS

Although the generalizability of the results of this project is limited by the participant recruitment and data collection methods (i.e., that the data arose from only 17 purposively recruited men who attend GCPs, who use drugs, and who engage in unsafe sex as part of their GCP-partying experience), these results nevertheless reveal an interesting narrative about this group of men who may be particularly vulnerable to HIV acquisition/transmission. What is important about such specified understandings of this group is that a large quantity of research has demonstrated that general population HIV interventions are not only a poor use of resources, but also are unlikely to effect desired population-level decreases in HIV transmission (see Aral, Lipshutz, & Douglas, 2007 and Fenton & Bloom, 2007, for further explanation about this point). The reason for this is that HIV is not a prevalent enough infection across North America and most of Europe to warrant general interventions. Instead, tailored and targeted interventions that address those groups that experience the greatest burden of HIV have consistently demonstrated successful public health outcomes. In other words, because HIV is not distributed evenly throughout the population at high enough levels, HIV prevention strategies yield their greatest impact when they are targeted specifically at those individuals who are most likely to encounter this infection—whether this is due to their sexual, occupational, or drug-using practices (Aral et al, 2007).

Bearing this in mind, these results highlight a few points of interest regarding GCP-related HIV prevention. First, these results indicate that some men seem to purposively consume drugs to explore their limits and then to excuse themselves for having done so. From an HIV prevention perspective, this could be important in relation to the underlying assumptions that guide substance-use-related HIV prevention strategies. Indeed, instead of assuming that all unsafe outcomes that follow substance use are accidental, it is important that HIV prevention workers determine whether this is actually the case among the individuals who they wish to target. This follows the work of Halkitis, Shrem, and Martin (2005), who found that crystal meth did not cause risky sexual practices, but rather men who frequently engaged in unsafe sex regardless of drug use were attracted to crystal meth. This raises an important point about the need for more in-depth research, which addresses substance use and unsafe sex.

In addition, these results raise the second point that HIV prevention workers working in STI clinics, public health or community-based workers who design HIV prevention interventions, or sociobehavioral researchers and students, should not accept the statement, “drugs made me do it” without further investigation. This does not mean, however, that such statements should be rejected; rather, the findings here simply indicate that, at times, for some individuals, this statement is used as an excuse and should be approached with a critical scepticism. For frontline clinicians and researchers who engage with individuals in a one-on-one basis about their behavior, this simply means that more questions are needed. Dig deeper to differentiate between genuine drug-induced behavior and which drugs are simply excusing.

Third, from a methodological standpoint, the results presented herein stress how important it is not to simply stop asking questions because the data correspond to socially held, socially acceptable, or personally held ideas that drugs cause unsafe behavior. Although our critical poststructuralistic theoretical position means that we most certainly do not recommend adopting bracketing as a method to address this potentially confounding factor, the findings here do support the idea that researchers need to be forthcoming in their assumptions and opinions about the topics that they investigate. This holds true for both qualitative and quantitative research. Nevertheless, it is important to remember that because of the small and focused nature of this research study, the foregoing results and suggestions should not be generalized to all drug and alcohol use. Indeed, they, as is always the case, should be used with extreme caution.

## FINAL REMARKS

In conclusion, the results of this research indicate that GCP-related drug use can be understood as a form of boundary play, a process of movement between the sober self and the intoxicated self. Indeed, it is possible to claim that drugs (including alcohol) permitted the 17 men in this study to deliberately explore their own boundaries and, later, to excuse themselves for having gone to extremes. As such, boundary play should not be confused with an intentional destruction of limits to the point of irrevocable negative changes. It is important to emphasize that drugs can be mechanisms, which individuals use to diminish problems that may result from actions undertaken while intoxicated. That is, boundary play is an attempt to remain safe despite having intentionally placed oneself in potentially unsafe conditions. Such results, while conflicting with the results of the 10 research studies that have addressed GCPs (because these other results, from a Deleuzian and Foucauldian perspective at least, seem to have concluded that drug use and its associated risky practices are the outcomes of underlying psychopathology), did not describe drug use as the result or outcome of underlying negative feelings. Rather, these practices were viewed as a method by which the research participants explored and experienced new sensations, and then excused this behavior *ex post facto.* In the language of this article, drug use thus permitted the participants to engage in boundary play. Nonetheless, returning to the fact that this finding arose from the interview data of only 17 participants, it is important that researchers further explore if/how this conceptual framework can be used to explain/predict the behavior of other groups that both consume drugs and engage in unsafe practices while intoxicated with these substances.

Declaration of Interest

The authors report no conflicts of interest. The authors alone are responsible for the content and writing of the article.

## GLOSSARY

Boundary play: Boundary play is the act of navigating edges, including the limits between sanity and insanity, legality and illegality, safety and danger, chaos and order. These boundaries are the dividing lines between two opposing states of existence, and playing with such boundaries is the act of approaching or treading on these lines. An essential component of such acts is that in flirting with danger, individuals must demonstrate the ability to safely navigate perilous edges, boundaries, or limits without suffering irreversible harm.
Deficit-based explanations: Deficit-based explanations are accounts of often socially censured behavior that describe these actions as the outcome of individual and societal shortcomings; for example, depression, isolation, and discrimination. From this perspective, the many benefits that arise from these socially marginalized behavior are overlooked and/or negated.
Gay circuit parties: Gay circuit parties can be described as multiday gatherings of thousands of gay and bisexual men in diverse venues that incorporate intricate light shows, unique dress codes, live disc jockeys, and various other performances. Moreover, these parties occur in the same place (city and venue) and at the same time of year (e.g., Thanksgiving).
